# Molecular Pathways Modulating Sensory Hair Cell Regeneration in Adult Mammalian Cochleae: Progress and Perspectives

**DOI:** 10.3390/ijms23010066

**Published:** 2021-12-22

**Authors:** Vikrant Rai, Shu Tu, Joseph R. Frank, Jian Zuo

**Affiliations:** Department of Biomedical Sciences, Creighton University School of Medicine, Omaha, NE 68178, USA; vrai@westernu.edu (V.R.); sxt735@case.edu (S.T.); joseph.frank@duke.edu (J.R.F.)

**Keywords:** hair cells, adult cochlea, regeneration, transcription factor, bioinformatics

## Abstract

Noise-induced, drug-related, and age-related disabling hearing loss is a major public health problem and affect approximately 466 million people worldwide. In non-mammalian vertebrates, the death of sensory hair cells (HCs) induces the proliferation and transdifferentiation of adjacent supporting cells into new HCs; however, this capacity is lost in juvenile and adult mammalian cochleae leading to permanent hearing loss. At present, cochlear implants and hearing devices are the only available treatments and can help patients to a certain extent; however, no biological approach or FDA-approved drug is effective to treat disabling hearing loss and restore hearing. Recently, regeneration of mammalian cochlear HCs by modulating molecular pathways or transcription factors has offered some promising results, although the immaturity of the regenerated HCs remains the biggest concern. Furthermore, most of the research done is in neonates and not in adults. This review focuses on critically summarizing the studies done in adult mammalian cochleae and discusses various strategies to elucidate novel transcription factors for better therapeutics.

## 1. Introduction

Noise-induced, drug-related, and age-related disabling hearing loss is a major public health problem. Per a World Health Organization report, they affect nearly 5% of the world population [1]. In non-mammalian vertebrates, sensory hair cell (HC) death induces the proliferation and trans-differentiation of adjacent supporting cells (SCs) into new HCs; however, this capacity is lost in juvenile and adult mammalian cochleae, leading to permanent hearing loss [2,3]. Currently, no biological approach or FDA-approved drug is available to treat disabling hearing loss or to regenerate the sensory HCs in mammalian cochleae. Thus, it is crucial to develop strategies or drugs to either prevent HC loss or promote regeneration in adult mammalian cochleae in vivo. HC regeneration can enhance the number of HCs in the cochlea via two processes: (1) mitotic regeneration, where a SC divides and then the daughter cells (one or both) transdifferentiate into HCs, or (2) direct transdifferentiation where HCs are regenerated via direct phenotypic conversion of SCs without undergoing mitosis [4]. Most of the studies on HC regeneration [3,5,6,7,8,9,10,11,12] have been done either in neonatal cochlear explants (ex vivo) or neonatal mice (in vivo) and only a few studies [5,8,9,10,11] have reported regeneration in juvenile and adult mice. Hearing matures around three weeks postnatally in mice. In comparison, during human fetal development hearing becomes mature by the late 2nd trimester and the fetus can hear during the 3rd trimester [13]. It is therefore critical to study HC regeneration in juvenile and adult mice to better understand which approaches are likely to restore hearing in humans. Further, the regenerated HCs in juvenile and adult mice are functionally immature and very few. Improved strategies are needed to increase the number of regenerated HCs and also to promote their maturation.

Recent studies suggest key roles for transcription factors (TFs) Atoh1 and Pou4f3 in HC regeneration and that Atoh1, in particular, is a master regulator of HC differentiation [14] and regeneration [3,8,10]. Further, enhanced regeneration of HCs via modulating the expression of Gata3, Pou4f3, p27^Kip1^ [8], Islet 1 (Isl1) [10], and Gfi1/Pou4f3 [15] with Atoh1 suggests that modulating multiple TFs in combination with Atoh1 is a good strategy to promote regeneration and increase the number of regenerated HCs. Targeting the Notch and Wnt signaling pathways, which are involved in HC development, can also lead to the regeneration of HCs from SCs [6,7,9,16,17]. These studies suggest that SCs have a limited regenerative capacity and that regeneration via transdifferentiation of SCs to HCs is possible, but a small yield of regenerated HCs and functional immaturity remain a major concern. Thus, there is a need to develop strategies to regenerate an increased number of HCs that are also functionally mature. This review focuses on recent literature in sensory HC regeneration in adult mammalian cochleae and briefly discusses molecular pathways, the role of TFs in regeneration, and the challenges and future perspectives of HC regeneration.

## 2. Targeting Signaling Pathways for Hair Cell Regeneration

**Figure 1:**
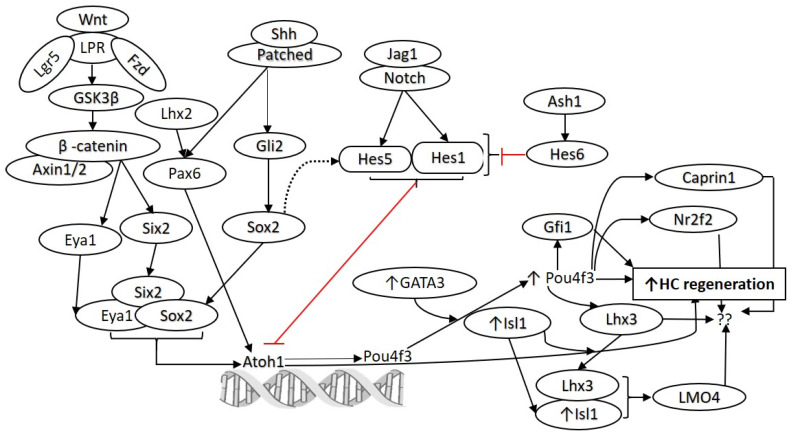
Molecular pathways involved in the development and regeneration of hair cells. Notch and Wnt signaling play a crucial role in the development and differentiation of HCs. Various studies have demonstrated that these pathways can be targeted for HC regeneration. Similarly, targeting Hes1, Gfi1, Pax6, Isl1, Pou4f3, Atoh1, and GATA3 to promote HC regeneration has been reported (as discussed in the text). However, there is a need to find additional candidate genes and transcription factors to promote HC regeneration. The network analysis on the published single-cell RNA-seq data (Yamashita et al. 2018) predicted other potential targets, including Lhx2, Hes6, Caprin1, Nr2f2, and Lhx3, which may be targeted alone or in combination to promote regeneration of HCs. Atonal BHLH Transcription Factor 1 (Atoh1), frizzled (Fzd), islet 1 (Isl1), jagged 1 (Jag1), lipoprotein receptor-related protein (LPR), POU Class 4 Homeobox 3 (Pou4f3), sonic hedgehog (Shh), Wingless-related integration site (Wnt). Black arrows show stimulatory while red arrows show inhibitory effect.

The Notch, Wingless-related integration site (Wnt), fibroblast growth factor (FGF), and sonic hedgehog (Shh) pathways are involved in the development and differentiation of HCs and are conserved between various species including zebrafish, birds, and mammals. The crucial role of these pathways in HC development and their conservation among the species are well characterized [18]. Briefly, Notch signaling regulates various cellular processes such as proliferation, differentiation, and cell death in a context-dependent manner. During HC development, Notch signaling is necessary and sufficient for regulating prosensory specification via lateral inhibition, and this process is mediated by its ligands which include jagged 1 (Jag1), Notch intracellular domain (NICD; which interacts with DNA-binding protein and core effector of the canonical Notch pathway, RBPjk), jagged 2 (Jag2), and delta-like 1 (Dll1). Wnt signaling (canonical and noncanonical) is involved in the maintenance of the progenitor cells, cell proliferation, cell fate determination/cell differentiation, and cellular polarization. The FGF signaling pathway plays a crucial role in the induction of the otic placode, development of the otic vesicle, regulation of inner ear morphogenesis, later stages of inner ear development, and HC formation. FGF signaling also helps in regulating the specification of prosensory cells and their differentiation into HCs and SCs during cochlear development [18]. The Shh signaling pathway is involved with regulating prosensory domain formation and auditory function [19], HC formation and differentiation [20], and the spatiotemporal pattern of HC differentiation via regulating the expression of Hey1 and Hey2 [21]. Involvement of the Notch, Wnt, bone morphogenetic protein (BMP), Shh, and fibroblast growth factor (FGF) pathways in the development, differentiation, maturation, and proliferation of HCs in zebrafish, birds, and mice provides strong evidence for significant conservation across species (Table 1). Wnt and Notch signaling play a crucial role in HC regeneration (Figure 1); however, the role of Shh and FGF signaling in regeneration remain unclear. The downstream signaling of these pathways regulates the expression of Atoh1, the master regulator of HC differentiation and regeneration [22]. Since ectopic overexpression of Atoh1 potentiates the regeneration of HCs, it is imperative to hypothesize that targeting these pathways and the genes and transcription factors regulating Atoh1 expression will be effective for the regeneration of HCs (Figure 1). Additionally, if regeneration of HCs from SCs follows development [23], targeting these pathways will be favorable for HC regeneration.

## 3. Targeting Notch Signaling for Hair Cell Regeneration

Both fate determination of prosensory epithelial cells into HCs and SCs through lateral inhibition and the prevention of SC to HC conversion during HC development are regulated by active Notch signaling stimulated by ligands on adjacent HCs [49]. Thus, inhibiting Notch signaling might lead to the transdifferentiation of SCs to HCs. Increased number of myosin-VII-positive outer hair cells (OHCs) in vitro with γ-secretase inhibitor, LY411575 suggests that Notch inhibition promotes regeneration via transdifferentiation of SCs. Treatment with LY411575 depleted the supporting cell population, but the number of inner hair cells (IHCs) remained unchanged [9]. In vivo studies with systemic injection of LY411575 (50 mg/kg body weight) for 5 days in noise-deafened mice (4 weeks) showed decreased noise-induced threshold shifts and an increased number of OHCs with apparently innervated stereociliary bundles [9]. A decreased expression of Hes5 and increased expression of Atoh1 associated with SC to HC transdifferentiation suggests an association of Notch inhibition with HC regeneration (apical to mid-apical turn). The regenerated HCs were lineage traced using Sox2-CreER; mT/mG mice with tamoxifen injection at postnatal day 21, confirming their SC origin [9]. The systemic injection of LY411575 was associated with toxicity and a lower dose was not therapeutically potent, however, local injection of LY411575 through the round window membrane showed significant transdifferentiation of SCs to HCs. Note that 3-million-fold higher concentrations of LY411575 (4 mM) than its IC50 (0.14 nM) were used [9]. Notch signaling in the cochlea becomes nonresponsive after the first postnatal week [50]. Additionally, >85% Sox2-CreER activity in SCs when induced at P21 compared to >50% when induced at P1 [51] makes Sox2 a good lineage marker when induction is performed in adult mice but not useful when induction is performed at birth. Additionally, a 92% reduction in the number of fate-mapped regenerated HCs in ROSA-NICD neonatal (P0-P1) mice with NICD (Notch) overexpression compared to the controls [52] supports the need for Notch inhibition in SC to HC transdifferentiation. However, enhanced proliferation of sensory HCs with transient coactivation of cell cycle activator Myc and Notch1 genes by injecting adenovirus (ad)-Myc/ad-Cre into the cochleae of 6-week-old Rosa-NICD transgenic mice seemed contradictory [11]. However, ad-Myc/ad-Cre injection could enable the SCs to proliferate and respond to Atoh1 and transdifferentiate to HC-like cells, which might be due to the differences between direct transdifferentiation vs. induced proliferative regeneration. Regeneration of HCs with sustained release of Hes1 siRNA nanoparticles (siHes1 NPs) in the cochleae of noise-injured adult guinea pigs supports Notch inhibition as a target for HC regeneration. The study reported limited recovery of auditory function over a nine-week follow-up period as well as HC regeneration, evident by the presence of both ectopic and immature HCs across a broad tonotopic range with siHes1 NPs. One of the major limitations of this guinea pig study is that no lineage tracing was performed to prove that newly regenerated HCs are derived from SCs. The advantage of using poly-lactic-co-glycolic acid (PLGA)-mediated siHes1 NPs delivery was its reversible modulation of Hes1 [5].

## 4. Targeting Wnt Signaling for Hair Cell Regeneration

Wnt signaling plays an important role in cochlear development and its role is context-dependent. Active canonical Wnt/β-catenin signaling is needed for the initial differentiation of HCs but not for maturation and maintenance. Overactive Wnt signaling results in HC proliferation and the formation of ectopic HCs during early embryonic development. This suggests that activating Wnt signaling will favor new HC formation through transdifferentiation or mitotic division [18,53]. The conserved nature of Wnt signaling among species (Table 1) and increased Wnt expression following HC loss appends the notion that increasing Wnt expression might promote SC-to-HC transdifferentiation. The association of activated Wnt/β-catenin signaling with SC proliferation, a transient proliferation of Lgr5+ SCs [54], and HC regeneration [55] support Wnt-mediated transdifferentiation. However, in these reports, it remains elusive whether the regeneration of HCs was due to either activated Wnt signaling or Sox2 haploinsufficiency (loss of one allele of Sox2 due to knockin of CreER in the Sox2 locus results in a haploinsufficient phenotype that produces extra inner hair cells during development and enhances regeneration). Later, Atkinson et al. showed that both β-catenin^GOF^ (gain of function) and Sox2 haploinsufficiency enhance mitotic regeneration in the apical turn whereas Sox2 haploinsufficiency-mediated mitotic regeneration extends into the middle and basal turns [34]. Jan et al. [56] using lineage tracing in P0-P3 Axin2^lacZ^ Wnt reporter mice showed that Wnt responsive Axin-2-positive tympanic border cells proliferate with Wnt activation and generate new HC- and SC-like cells both in vitro and in vivo and can act as a precursor to sensory epithelial HCs. These studies suggest that activation of Wnt signaling in neonatal mice potentiates regeneration of HCs, however, no study has shown increased regeneration by activating Wnt signaling in adults.

## 5. Combinational Approaches for Hair Cell Regeneration

The role of Wnt activation in SC proliferation and Notch inhibition in transdifferentiation of SCs to HCs is evident by the above studies. Additionally, Wnt activation alone fails to regenerate significant amounts of new HCs in adult mammals, and Notch inhibition alone regenerates HCs at the cost of SCs, resulting in the death of regenerated HCs. Thus, maintaining the population of SCs via proliferation along with SC-to-HC transdifferentiation might enhance the regeneration process by sufficing the SC population for differentiation. Ni et al. reported that Wnt activation with 6-Bromoindirubin-3′-oxime (BIO), a glycogen synthase kinase 3 β (GSK3β) inhibitor, followed by Notch inhibition with DAPT, a γ-secretase inhibitor, preserves the Lgr5+ SC number and strongly promotes the mitotic regeneration of new HCs in both normal and neomycin-damaged cochlear explants (P1; C57/BL6 mice) [17]. Similar findings were reported by Wu et al. [57] by simultaneously inhibiting Notch signaling with DAPT and activating Wnt signaling with Wnt agonist QS11. The first study used the explants from P1 mice while the second study showed it in the utricle of neonatal mice, which itself has some regenerative capacity. Since Notch and Wnt signaling have a reciprocal relationship during HC development, a combined modulation of Notch and Wnt signaling might be a better approach for regeneration. Increased HC regeneration using Notch inhibition followed by Wnt activation in adult and neonatal mouse cochleae has been reported [17,58,59]. Romero-Carvajal et al. highlighted the role of interactions between Notch and Wnt signaling for the regeneration of HCs in zebrafish and demonstrated that inhibition of Notch signaling mimics the expression changes observed during endogenous regeneration [60].

Targeting multiple pathways and factors involved in HC development and insight from their involvement in other regenerative systems could be a promising approach to enhance HC regeneration in the cochlea. Clonal expansion of Lgr5+ SCs isolated from a neonatal cochlea showed that in a matrigel-based 3D culture system, a mixture of growth factors (including epidermal growth factor, basic fibroblast growth factor, and insulin-like growth factor 1), GSK3β inhibitor, histone deacetylase (HDAC) inhibitor, and Notch inhibition led to transcriptional activation, proliferation, and differentiation of SCs [6]. The addition of a stable form of vitamin C and transforming growth factor β (TGF-β) receptor (Alk5) inhibitor individually resulted in increased SC expansion by 2- to 3-fold, and the addition of small molecules in combination with growth factors increased the expansion of Lgr5+ SC numbers by >2000-fold compared to growth factors alone. The addition of these small molecules and γ-secretase inhibitor resulted in the expansion and differentiation of Lgr5+ SCs from a single mouse cochlea to nearly 11,500 HCs in culture organoid. The newly generated HCs in the organoid were myosin VIIa+ cells containing CtBP2+ ribbon synapse-like puncta in the basal region and actin-rich protrusions within the inner lumen. The colonies were both prestin-positive and negative; prestin-negative cells were vesicular glutamate transporter 3 (vGlut3)-positive, reflecting the gene expression of terminally differentiated OHCs and IHCs, respectively. The combination of these small molecules generated a higher number of new HCs in culture and in neonatal cochlear explants compared to the adult mouse Lgr5+ cells. There was no significant difference across the ages. Similarly, the clonal expansion and differentiation of adult human inner ear tissue was also limited [6]. The results of this study suggest that targeting a single gene, TF, or pathway may not be sufficient for HC regeneration and that there is a need for multidimensional approaches to promote transdifferentiation and regeneration. A recent study demonstrated the conversion of mouse embryonic fibroblasts, adult tail-tip fibroblasts, and postnatal supporting cells into induced hair cell-like cells (iHCs) showing HC-like morphology, transcriptomic and epigenetic profiles, electrophysiological properties, and mechanosensory channel expression using a combination of four transcription factors, Six1, Atoh1, Pou4f3, and Gfi1 [61]. Similar results of an increased SC to HC conversion by modulating the expression of p27^kip1^, GATA3, and Pou4f3 in combination with Atoh1 were reported in adult mouse cochleae by Walters et al. [8]. These studies support the notion of using a combinational approach to promote HC regeneration via direct reprogramming. Notch, Wnt, and other signaling pathways play a crucial role in the development and proliferation of HCs and are conserved among species including zebrafish, birds, and mice (Table 1); targeting these pathways concomitantly might enhance HC regeneration in adult mammals (Figure 1) [34].

## 6. Modulating Transcription Factors for Hair Cell Regeneration

Transcription factors (TFs) are the proteins initiating and regulating transcription of target genes by binding to their specific regulatory DNA sequences. TFs play a crucial role in the proliferation, differentiation, and survival of HCs. The role of TFs such as Pax2, Sox9, Nor-1, Gbx-2, Neurod1, Neurog1, Fkh10, Tbx1, Brn4, Gata3, Sox2, Atoh1, Six1, Isl1, Pou4f3, Gfi1, and their interactions with cellular and molecular signaling pathways in prosensory cell specification, development, and fate determination of HCs in the inner ear and vestibular apparatus have been described by other groups [62,63]. Since TFs play an important role in HC development and fate determination, investigating their role will help not only in understanding HC development but also in modifying regeneration strategies for improved outcomes. TFs, individually or in combination, play a crucial role in the regeneration of other organ systems [64,65,66]. Thus, it is necessary to investigate the TFs which might be capable of potentiating the regeneration process in the cochlea and to understand the underlying mechanisms. Costa et al. studied the role of three TFs, namely, Gfi1, Atoh1, and Pou4f3 (GAP) in cell fate determination, and reported that GAP (Figure 1) can induce direct genetic reprogramming of progenitors towards an HC fate, both in vitro and in vivo in the chicken embryo [15]. Another study reported that overexpression of Prox1 suppresses Atoh1 and Gfi1 expression and antagonizes the differentiated HC phenotype; thus, Prox1 inhibition with Atoh1 upregulation might result in a more complete phenotypic conversion [67]. These studies were performed in the embryonic stage and whether SCs can be transdifferentiated to HCs, postnatally or in adults, cannot be determined. These studies allude to Atoh1 as a common denominator target for HC regeneration.

**Figure 2:**
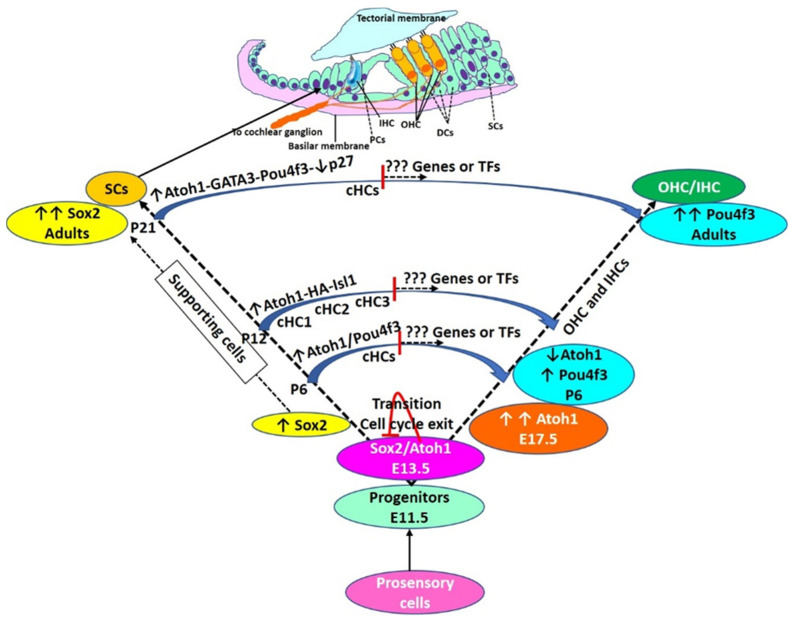
Hair cell development and regeneration: During the embryonic stage of HC development, Atoh1 expression increases, reaches a maximum (E17.5), and then declines (P6). The decline in Atoh1 is associated with increasing levels of Pou4f3 which remain high in adult HCs. During the embryonic stage, autoregulation of Atoh1, Sox2, and cell cycle exit. Some cells have high levels of Sox2 without Atoh1 expression and these cells are deemed to be SCs. Transdifferentiation of supporting cells (SCs) to HCs is mediated by overexpression of transcription factors (TFs) as discussed in the text and shown here. However, TF-mediated transdifferentiation is not complete and converted HCs (cHCs) remain immature. cHCs show only some features of mature HCs (shown in the middle of the trajectory of conversion), and thus, there is a need to investigate novel targets to push these partially converted cells to mature HCs. Pillar cells (PCs), Deiters’ cells (DCs), inner hair cells (IHC), outer hair cells (OHC), postnatal day (P). cHCs are the cells evaluated for their transcriptome by single cells RNA sequencing by Yamashita et al., 2018.

Atoh1 is a master regulator of HC differentiation that is conserved among fish (ortholog atoh1a), birds, and mice (Table 1). Liu et al. [12] investigated the effect of ectopic expression ofAtoh1 on regeneration using EGFP reporter mice and reported that ectopic expression of Atoh1 induces the conversion of mouse cochlear SCs (pillar and Deiters’ cells; PCs and DCs) to immature HCs, and that this conversion is age-dependent. Ectopic Atoh1 expression was effective in converting PCs and DCs to HCs at neonatal and juvenile ages, but it was insufficient for adult mice. It was found that newly formed HCs reside in the OHC region and survive for 2 months, and that heterogeneity in the reprogramming efficiency among individual Atoh1+ PCs and DCs exists during the conversion process. These studies suggest that transcriptional reprogramming affects the HC phenotype and thus might favor regeneration, but the limitation is that these results were shown at neonatal and juvenile ages and Atoh1 overexpression alone was not effective in adult mice. To address this issue, Walters et al. [8] investigated the role of various TFs in adult mice and reported that ectopic Atoh1 overexpression with p27^Kip1^ deletion circumvents this age-related decline in Atoh1 responsiveness and leads to transdifferentiation of SCs to HCs in mature mouse cochleae after noise damage. Further, upregulation of an Atoh1 cofactor, GATA3, which is lost from SCs with aging, was associated with p27^Kip1^ deletion. Overexpression of POU4F3 alone promoted the conversion of SCs to HCs to a greater degree than Atoh1 alone, and overexpression of Atoh1 combined with POU4F3 or GATA3 resulted in increased conversion of SCs to HCs compared to Atoh1 alone in adult mice [8]. The study concluded that the mature PCs and DCs, which are typically nonresponsive to Atoh1, can be made to respond to ectopic Atoh1 via modulation of additional TFs such as p27^Kip1^, GATA3, or POU4F3 (Figure 1 and Figure 2). However, the converted HCs were examined only at 3 and 12 weeks following tamoxifen injection, and the long-term survival of these cells was not extensively evaluated [68].

Increased conversion of SCs to HCs by activating Atoh1 conditionally with tamoxifen and 51 differentially expressed TFs between endogenous OHCs, SCs, and converted HCs (cHCs) in the adult cochlea with bulk-RNA sequencing, single-cell RNA sequencing, and single-cell RT-PCR reported by Yamashita et al. [10] supports targeting Atoh1 for regeneration and warrants research for the role of these TFs in HC regeneration. Additionally, a greater number of cHCs with combined overexpression of Atoh1 and Isl1 (one of the differentially expressed TFs in RNA-seq analysis) compared to overexpression of Atoh1 alone both ex vivo and in vivo supports the hypothesis that the conversion process can be pushed further to get a greater number of cHCs by targeting multiple TFs. However, the study only reported the cHCs at two-time points of conversion and the cHCs were not functionally mature (Figure 2).

Prolonged constitutive ectopic Atoh1 expression with tamoxifen using a Cre-inducible mouse might be a cause of immaturity in cHCs because continued Atoh1 expression does not correlate with endogenous HC development [69]. Controlled activation of Atoh1 using tetracycline (e.g., dox)-inducible systems is difficult to achieve in juvenile or adult mice due to the long-term residual activity of tetracycline in the cochlea [70,71]. However, these studies give hope that a greater number of regenerated HCs can be achieved at an adult age. Further, the role of TFs Insm1 [72] and Ikzf2 [73] in the fate determination and functional maturation of OHCs suggests the possibility of other TFs having critical regulatory roles in the regeneration of HCs. Recently, the role of TUB and ZNF532 in promoting Atoh1-mediated hair cell regeneration in mouse cochleae was reported by Xu et al. [74]. Thus, investigating novel TFs and targets upstream or downstream of Atoh1 is warranted, and modulating their expression alone or in combination could provide better results (Figure 1).

## 7. SC Subpopulations and Hair Cell Regeneration

Transdifferentiation of SCs to HCs has been postulated as the main strategy to regenerate HCs, and most of the studies discussed above have targeted various signaling pathways and TFs in order to do so. However, the debate on which subtype of SCs is more prone to transdifferentiation still exists in the field. Walters et al. [8] reported the unresponsiveness of mature PCs and DCs to Atoh1 and, together with other evidence, concluded that responsiveness to Atoh1 varies across SC subtypes. Thus, it is important to investigate the differential responsiveness of SC subtypes to TFs to achieve a greater number of functionally matured HCs through regeneration. Recently, Hoa et al. [75] reported that adult cochlear SCs are transcriptionally different from perinatal SCs by conducting single-cell RNA-Seq on FACS-sorted GFP expressing adult cochlear SCs from Lfng^EGFP^ adult mice. The study found two different subpopulations of SCs (SC1 and SC2). The SC2 subpopulation expresses transcripts associated with S phase (Mcm4) and G2/M phase (Birc5, Cdk1, Mki67). Cheng et al. [76] also reported differential expression of various cell cycle and signaling pathway genes and TFs in Sox2+ SCs at four different postnatal ages suggesting the existence of age-related transcriptomic landscape changes. The different transcriptomic landscape of the perinatal and postnatal SCs found in this study might be the reason for the differential responsiveness of adult SCs in the study by Walters et al. [8]. Further, the findings of strong expression of the SC genes involved in pathways regulating the cell cycle [75] suggest that these pathways may be targeted to potentiate the transdifferentiation of SCs to HCs by forcing the SCs out of quiescence. This notion is supported by DCs and PCs contributing more to the spontaneously regenerated HCs but inner phalangeal (IPhs) and inner border (IBs) cells having similar regenerative capacity in neonatal mice [77]. This differential response may be because PCs and DCs lose the cell cycle inhibitor p27^Kip1^ during postnatal development and are capable of mitotic HC regeneration. These findings are supported by a previous study in juvenile mice where ectopic expression of Atoh1 induces SC-to-HC conversion and the newly regenerated HCs are mainly from PCs and DCs [12]. Later, however, a higher, faster, and more complete conversion rate of IBs and IPhs compared to DCs or PCs to IHC-like cells was observed in vivo, as evidenced by straight line-shaped stereociliary bundles, expression of Fgf8 and otoferlin, and by ectopic Atoh1 expression [78]. The study also reported that the conversion rate gradually increases from neonate to adult ages in mice. Differential regenerative capacity of SCs might be due to changing Sox2 expression over time. Changing Sox2 expression was reported by Kempfle et al. [79] suggest that Sox2 is expressed in prosensory cells of the cochlea at E13, in the developing sensory epithelium at E15 and E18, in newly formed IHCs at E15, and its expression continues in newly formed IHCs and OHCs at E18 until P0 and becomes undetectable at P2. Sox2 is strongly expressed in SCs at E18 and continues to be expressed in SCs at P2. Sox2 is necessary for differentiation as deletion of Sox2 at E16 led to no further differentiation of HCs.

The Lgr5+ subtype of SCs has been an attractive target for HC regeneration. Kuo et al. reported an increased number of regenerated HCs via transdifferentiation of Lgr5+ SCs by ectopically co-expressing a constitutively active form of β-catenin and Atoh1 in Lgr5+ cells of the neonatal cochlea. This study suggests that combining proliferation and differentiation of Lgr5+ SCs by coactivating β-catenin and Atoh1 acts synergistically to enhance the process of regeneration, yielding an increased number of regenerated HCs [16]. Although the tamoxifen induction was done at a neonatal age, the study reported the HCs had an adult phenotype. Recently, Zhang et al. reported that activating Frizzled-9 (Fzd9)-positive cells in neonatal mouse cochleae leads to regeneration of a similar number of HCs. Lineage tracing of the tamoxifen-induced cells showed that inner phalangeal cells (IPhCs), inner border cells (IBCs), and third-row Deiters’ cells (DCs) were both Fzd9+ and Lgr5+, while pillar cells are Lgr5+ only [7]. The study concluded that the Fzd9+ cells have a similar capacity for HC regeneration, proliferation, and differentiation compared to Lgr5+ cells. These results demonstrate the potential of targeting Notch and Wnt signaling for HC regeneration; however, there is a need to translate these findings to pre-clinical trials and future studies are warranted. Collectively, these studies suggest there is heterogeneity and a changing transcriptomic landscape of SCs over time. Additionally, there are no known differences among different mammalian species in HC regeneration relative to the timing of HC development. Thus, the strategies and timing of manipulating SCs for regeneration are of the utmost importance and warrants further investigation.

## 8. Finding Additional Transcription Factors as Novel Targets for Hair Cell Regeneration

Atoh1 regulates HC development and differentiation, and overexpression of Atoh1 regenerates HCs from SCs; however, the newly regenerated HCs are fewer, short-lived, and not functionally mature, as evidenced by the absence of prestin, the marker for OHC maturation [8]. Thus, the consensus is to find novel targets upstream or downstream of Atoh1 whose modulation can potentiate regeneration so that increased numbers of functionally mature HCs can be achieved. This notion is supported by the fact that co-activation of Atoh1 with Pou4f3 [8], with Isl1 [10], and with both Pou4f3 and Gfi1 combined [15] yielded a greater number of HCs compared to activation of Atoh1 alone. This suggests that either post-transcriptional modification of Atoh1 targets, Atoh1 itself, or epigenetic regulation of Atoh1 and its targets regulate the expression of various target genes and TFs, and thus HC regeneration. To investigate the direct targets of Atoh1, Cai et al. [80] carried out RNA-seq profiling of purified Atoh1 expressing HCs from neonatal mouse cochleae and identified >600 enriched transcripts with 233 HC genes directly regulated by Atoh1. Atoh1 regulation was verified by the presence of Atoh1 binding sites in the regulatory regions of these genes and by the cerebellum and small intestine Atoh1 ChIP-seq analysis. Anxa4, Rasd2, Rbm24, Srrm4, Chrna10, Mgat5b, Mreg, Pcp4, Scn11a, and Atoh1 were found to be direct targets of Atoh1. The expression of Anxa4, Rasd2, Rbm24, and Srrm4 was completely downregulated within 24 h after knocking out Atoh1, but the expression of Chrna10, Mgat5b, Mreg, Pcp4, and Scn11a were not affected. In the context of epigenetic regulation of Atoh1, Jen et al. reported that the mouse vestibular apparatus has greater Atoh1-mediated regeneration compared to the cochlea due to greater chromatin accessibility [81]. These findings suggest that differential efficiency of Atoh1-mediated regeneration is due to the non-availability of open chromatin in the cochlea and warrants further research using ATAC-seq (Assay for Transposase-Accessible Chromatin using sequencing) and ChIP-seq to unravel the epigenetic regulation and to identify additional targets for regeneration.

## 9. Investigating Epigenetic Regulation of Hair Cell Development and Regeneration

Coordinated and structured gene expression is a must for cellular development, differentiation, and survival, and epigenetics plays a crucial role in regulating gene transcription and expression. Post-translational histone (basic proteins in the cell nucleus) modification mechanisms include methylation (addition of a methyl group), acetylation (addition of an acetyl group), phosphorylation, and ubiquitination, which regulate chromatin architecture and gene expression. Methylation reduces gene expression by impairing the binding of transcriptional activators whereas acetylation increases gene expression by transcription activation. Histone acetylation is regulated by histone acetyltransferases (HATs) and histone deacetylases (HDACs); methylation and demethylation are regulated by histone methyltransferases (HMTs), DNA methyltransferase (DNMTs), and histone demethylases. Epigenetics play a role in hereditary or syndromic hearing loss by regulating gene expression and HC development [82,83,84]. Stojanova et al. [85] investigated the epigenetic regulation of Atoh1 and found that progression of Atoh1 expression from poised, to active, to repressive marks is controlled by dynamic changes in histone modifications via methylation and acetylation (H3K4me3/H3K27me3, H3K9ac, and H3K9me3) and correlates with the onset and subsequent silencing of Atoh1 expression in HCs during the perinatal period. The study reported that during HC differentiation, increased Atoh1 expression correlates with increased levels of H3k9ac (H3K9 histone acetylation) and that during HC maturation decreased levels of Atoh1 correlate with decreased levels of H3K9ac and increased levels of H3K9me3. Further, increased expression of HC-related genes and proteins in mouse utricle sensory epithelia-derived progenitor cells with DNMT inhibitor 5-azacytidine suggests an important role for epigenetics in HC differentiation [86]. This notion is also supported by the recent report by McLean et al. [6] where an HDAC inhibitor was used for the regeneration of HCs. However, Layman et al. [87] reported that suberoylanilide hydroxamic acid (SAHA, an HDAC inhibitor) does not affect regeneration in adult cochleae but instead activates pro-survival pathways via regulating the acetylation status of transcription factors and controls the transcriptional activation of pro-survival pathways in response to ototoxic insults. These surprising results suggest that HDAC inhibitors cannot effectively modulate the already fixed epigenetic landscape of adult cochlear SCs and are thus ineffective in reprogramming. HC fate determination and development are highly regulated processes under the influence of various TFs and gene expression, and expression of this transcriptomic landscape changes over time [83,84], with a dramatic change in the transcriptomic landscape between post-natal day (P)5–P7. Thus, investigating the epigenetic regulation of TFs and genes involved in HC development and rescripting the genetic landscape may provide insights to promote HC regeneration.

## 10. In Silico Approaches to Finding Novel Gene Targets

In silico analysis and the use of the wealth of bioinformatics applications for the acquisition of biological data and data mining have changed the paradigm of research in the field of basic and applied science. In the auditory field, regeneration of HCs deals with the modulation of genes and TFs, thus we can analyze the available databases to uncover better targets to modulate and potentiate the process of regeneration. The binding of TFs to their corresponding TF binding sites (TFBSs) is key to transcriptional regulation. Because information on experimentally validated functional TFBSs is limited, there is a need for the prediction of TFBSs for gene annotation. TFBSs are generally recognized by scanning a position weight matrix (PWM) against DNA sequences using one of several available computer programs. There are also several curated databases of PWMs, applicable to a wide range of species, including the commercial TRANSFAC database [88] and the open-access JASPAR database [89]. Other recent databases include the HOMER motif (http://homer.salk.edu/homer/motif (accessed on 6 June 2020)) HOCOMOCO [90], and CIS-BP [91]. There is a particularly useful program, the Cytoscape plugin iRegulon [92], which can discover master regulators from co-expressed gene sets. Additionally, the methods of inferring co-expression networks from single-cell RNA-seq data and workflow, such as single-cell regulatory network inference and clustering (SCENIC) [34], have been developed to exploit the genomic regulatory code (regulon), guiding the identification of master TFs and revealing different cell states. Such predictions on the master regulators of different cell types/states would be valuable to improve the conversion efficiency from SCs to HCs. Further, network analysis using these tools might predict the master regulators whose modulation, either alone or in combination with other TFs, may promote regeneration.

The network analysis done on an scRNA-seq data of cHCs [10] predicted Lhx3, Six2, Hes2, Irf6, Hes6, and Ikzf2 along with Atoh1 as candidate targets to modulate. Ikzf2 has recently been shown to be crucial for OHC fate and maturation, as prestin and oncomodulin expression is lost in Ikzf2-mutant mice [73]; contrarily, overexpression of Ikzf2 in IHCs leads to downregulation of IHC genes and upregulation of OHC genes. Transformation of adult cochlear SCs into prestin-positive OHCs with concurrent stimulation if Athoh1 and Ikzf2 supports the role of Ikzf2 in transdifferentiation [93]. Hes6 has also been implicated in the differentiation of mammalian HCs [94]. Interestingly, identification of the TFs such as Hes2, Hes6, Irf6, and Atoh1, which have roles in neural development and differentiation [95,96,97,98], by our network analysis suggests the feasibility and promising role of using bioinformatics to identify novel targets. Another TF identified in our network analysis, Six2, appears to play a role in regeneration in the mammalian kidney, as it is expressed in self-renewing progenitor cells within this organ [99]. These results suggest that the TFs identified via bioinformatics analysis of cochlear scRNA-seq data play a role in the regeneration and development of other organ systems and hence should be investigated for cochlear HC regeneration, and that further bioinformatics analysis of the existing cochlear scRNA-seq or ATAC-seq data is warranted.

## 11. Conclusions

Modulating the expression of signaling pathways and genes involved in sensory HC development, as discussed above, has given promising results in adult cochlear HC regeneration; however, the small number and functional immaturity of regenerated HCs remain a challenge. Targeting multiple factors has improved the outcome, but there is still a need to investigate additional targets and to form novel strategies to promote HC regeneration in adult mammals and then to translate these to clinics. The downstream targets of Atoh1 and Pou4f3 might be viable targets for HC regeneration. If regeneration follows development, unraveling the sequential targets for regeneration is of the utmost importance. Similarly, the role of many TFs such as Lhx3, caprin1, Nr2f2, Lmo4, and others in the regeneration process has not been investigated. Analyzing the existing cochlear data using bioinformatics tools investigating endogenous regeneration in zebrafish and birds might give the hearing field an overview and insight into what factors remain to be modified to regenerate HCs that are greater in number and functionally mature. Taken together, investigating the genes and TFs which either alone or in combination can potentiate the transdifferentiation of SCs to HCs should be the focus of current research for better therapeutics.

## Figures and Tables

**Table 1:** Comparative summary of signaling pathways, genes, and transcription factors involved in the development, differentiation, proliferation, and regeneration of HCs among species. ATOH1 (atonal BHLH Transcription Factor 1); Shh (sonic hedgehog); HC (hair cell); SC (supporting cell); OHC (outer hair cells); BP (basilar papilla); FGF (fibroblast growth factor); Fgfr (fibroblast growth factor receptor).

Target	Zebrafish	Birds	Mice	Inference
Notch Atoh1	atoh1a is expressed in all differentiating hair cells [24] Addition of exogenous atoh1a mRNA results in HC overproduction [25]	Atoh1 is immediately upregulated in Sox2+ SCs of the avian BP following HC loss [26]; Atoh1 protein expression is not detectable in mature HCs or SCs in the absence of damage [26]	Math1-null mice fail to develop cochlear and vestibular HCs [27] Atoh1 overexpression can convert SCs into HC-like cells in neonatal mouse cochleae [12]	Atoh1 modulation promotes regeneration in juvenile and adult mice, hence being a potential therapeutic target
Notch Hes5	Does not affect atoh1a expression [28] Hes5 morphants do not generate supernumerary HCs [28]	Notch signaling activates Hes5 expression which inhibits hair cell fate [29] Hes5 is downregulated in SCs following HC damage and loss [30,31]	Beginning at postnatal ages, Hes5 is restricted to supporting cells [32], Hes5 deletion results primarily in supernumerary OHC formation, but also some supernumerary IHC formation [32]	Hes5 inhibition might be a therapeutic target in HC regeneration.
Notch Hey1/Hey 2	Hey1 is downregulated in hair cells [24]	Hey1 and Hey2 are activated by Notch signaling in the basilar papilla and inhibit HC fate [33]	Hey1 and Hey2 negatively regulate Atoh1 to prevent premature HC differentiation [21] Exogenous Shh increases Hey1 and Hey2 mRNA levels in cochlear explants [21]	Hey1/2 inhibition may help regenerate HC-like cells to adopt a more HC-like phenotype.
Wnt β-catenin	Wnt/β-catenin inhibition in embryonic zebrafish reduces proliferation of sox2+ SCs in the developing neuromast [34]; Wnt/β-catenin is upregulated in SCs following HC loss [35] but is not sufficient for regeneration	Increases the proliferation of SCs following HC damage and regulates the number of HCs that form in the embryonic basilar papilla [36], Forced expression of β-catenin and Wnt3a results in the formation of ectopic sensory patches within the embryonic basilar papilla [37]	Activation of Wnt/β -catenin results in proliferation of Sox2+ SCs [17]; Lgr5+ SCs exhibit increased proliferation and differentiation into HCs in vivo in mice which overexpress β-catenin and Atoh1 [16]	β-catenin is a key therapeutic target for expansion of the HC progenitor pool. Wnt/β-catenin is conserved between species and plays a role in HC development and proliferation
Shh	Modifying hedgehog signaling interferes with axial patterning of the zebrafish otic vesicle [38]	Ectopic Shh signaling induces apical hair cell identities in the basal and middle regions of the avian basilar papilla [39]	Constitutive activation of Shh signaling hinders HC differentiation in developing murine cochleae [20] Inhibition of hedgehog signaling in cochlear explants results in an expanded sensory domain and formation of ectopic hair cells [19]	Modifying Shh signaling does not seem to be an effective strategy to promote regeneration
FGF	Fgf signaling is required for Atoh1 expression and hair cell development [25] During development, weak Fgf inhibition expands the sox2+ prosensory domain while strong Fgf inhibition reduces the sox2+ prosensory domain [40] While Fgf inhibition hinders HC differentiation, by expanding the sox2+ prosensory domain, it ultimately results in the overproduction of hair cells Fgfr3 knockout results in supernumerary HC formation [40] Fgf8 knockout results in reduced HC formation [40] Fgf3 overexpression results in reduced HC number [40]	Inhibition of FGF signaling in E5-E9 chicks results in overproduction of HCs through non-proliferative mechanisms. FGF inhibition increases the number of Sox2+ HCs in the embryonic basilar papilla, suggesting that the formation of extra hair cells is due to transdifferentiation [41] Fgfr3 is restricted to supporting cells in the mature basilar papilla [42] Fgfr3 expression is downregulated in the mature basilar papilla following damage to hair cells [42]	Fgfr1 hypomorphs lack 3rd-row OHCs [43] Fgfr1 plays a role in prosensory specification [44], In the embryo, Fgfr3 is expressed in the area of the cochlear duct that gives rise to pillar cells, OHCs, and Deiter’s cells, but Fgfr3 is confined to pillar cells by birth [45], Activation of Fgfr3 with Fgf17 inhibits OHC differentiation without affecting IHCs [46], Pan Fgf inhibition decreases expression of Atoh1 in murine cochlear explants [47], Fgfr3-/- mice lack a row of pillar cells, but have an ectopic additional row of Deiters cells and an additional row of OHCs which appear to have normal bundle morphology [48]	FGF signaling seems to be important signaling to modulate to promote HC regeneration, however, the results seem to be receptor-specific and different receptors have different effects of modulating FGF signaling

## Data Availability

Not applicable.
